# Mediating and Moderating Role of Depression, Conduct Disorder or Attention-Deficit/Hyperactivity Disorder in Developing Adolescent Substance Use Disorders: A Population-Based Study

**DOI:** 10.1371/journal.pone.0157488

**Published:** 2016-06-13

**Authors:** Kouichi Yoshimasu, William J. Barbaresi, Robert C. Colligan, Robert G. Voigt, Amy L. Weaver, Slavica K. Katusic

**Affiliations:** 1 Division of Epidemiology, Department of Health Sciences Research, Mayo Clinic, Rochester, Minnesota, United States of America; 2 Division of Developmental Medicine, Department of Medicine, Boston Children's Hospital, Boston, Massachusetts, United States of America; 3 Department of Psychiatry and Psychology, Mayo Clinic, Rochester, Minnesota, United States of America; 4 Department of Pediatrics, Baylor College of Medicine, Houston, Texas, United States of America; 5 Division of Biomedical Statistics and Informatics, Department of Health Sciences Research, Mayo Clinic, Rochester, Minnesota, United States of America; 6 Department of Hygiene, School of Medicine, Wakayama Medical University, Wakayama City, Japan; Harvard Medical School, UNITED STATES

## Abstract

**Objective:**

To evaluate the mediating/moderating effects of common internalizing /externalizing disorders on the association between ADHD and adolescent substance use disorders (SUD) in a population-based birth cohort.

**Methods:**

Among 5718 children in the birth cohort, 343 ADHD incident cases and 712 matched controls were identified. Psychiatric diagnoses prior to age 19 were classified into DSM-IV categories. The association between ADHD and SUD was summarized (hazard ratios (HR), 95% CI). The effect of depression, CD/ODD, anxiety was evaluated separately.

**Results:**

Assessment of the joint effects of ADHD and each psychiatric disorder did not support a moderating effect of these disorders on SUD on additive scale. However, the association between ADHD and SUD was partially explained by a mediating role of these psychiatric disorders.

**Conclusion:**

For clinicians our results emphasize that depression (or CD/ODD) confers greater risk for SUD than ADHD alone. Early detection/treatment of SUD among adolescents with depression (or CD/ODD) is crucial regardless of ADHD.

## Introduction

Attention deficit/hyperactivity disorder (ADHD), characterized by developmentally inappropriate levels of inattentiveness, impulsivity, and/or hyperactivity, is often accompanied by psychiatric disorders, such as mood disorders, anxiety disorders, conduct disorder (CD), and oppositional defiant disorder (ODD) [1–4]. In a previous study, we identified a broad range of psychiatric comorbidities, including both externalizing and internalizing disorders, in childhood ADHD cases from a population-based birth cohort [5]. Substance use disorder (SUD) among adolescents with ADHD is often strongly connected with such internalizing and externalizing comorbidities [6–9].

A recent meta-analytic review emphasized that the association between ADHD and SUD cannot be adequately addressed unless comorbidity with CD/ODD is considered [10]. Furthermore, depression and anxiety disorders have also been shown to be associated with both ADHD and SUD [5, 11]. For example, a systematic review of adolescent patients hospitalized for self-harm showed that depression, anxiety disorders, ADHD, CD and alcohol misuse were very common [12]. These findings suggest that among a variety of mental disorders associated with ADHD, CD/ODD (typical externalizing disorders) and depression and anxiety (typical internalizing disorders) might exert some moderating or mediating effects on the association between ADHD and SUD. Internalizing and externalizing classification of these disorders have been consistent in previous studies [13, 14]. Therefore, evaluating the relationships among these disorders is vital for effective clinical management.

There are some epidemiological studies evaluating the clinical associations between externalizing disorders and ADHD on SUD [15,16], but there are very few epidemiological studies examining the effects of internalizing disorders on ADHD and SUD in childhood and adolescence. Vidal et al. [17] showed that offspring of patients with SUD were likely to suffer from depression, ADHD, and SUD. In addition, child anxiety was shown to play a moderating role on outcome after multimodal treatment of ADHD [18]. Thus, further study of the moderating and mediating mechanisms that explain risk for early substance use and abuse in children with ADHD is warranted [15]. A moderating variable is one that influences the strength of the association between ADHD and SUD, whereas a mediator is considered to be an explanatory link in the causal pathway between ADHD and SUD [19].

Given the serious burden of SUD on society, families, and adolescents’ quality of life (QOL) [20], early detection and treatment are crucial. The present study evaluated the mediating and moderating effect of depression, CD/ODD, and anxiety on the association between ADHD and SUD among adolescents in a population-based birth cohort.

## Method

### Study setting and data source

This study was conducted in Rochester, Minnesota, located 90 miles southeast of Minneapolis-St Paul, the closest major urban center. According to 1990 census data, when subjects in our study were school-aged children, Rochester had 70,745 residents: 96% white, 72% ≤45 years of age, and primarily middle class. The demographic characteristics of Olmsted County (Rochester Standard Metropolitan Statistical Area) residents resembled those of the US white population during the timeframe relevant to this study [21].

The capacity for population-based epidemiologic research on psychiatric comorbidities of ADHD in Rochester is the result of a unique set of circumstances. First, Rochester is relatively isolated in southeastern Minnesota, and as a result, virtually all medical care is provided locally by Mayo Clinic (MC) and Olmsted Medical Center (OMC) and their three affiliated hospitals. Through the Rochester Epidemiology Project, which includes a unique medical record-linkage system of Rochester and Olmsted County residents, all diagnoses and surgical procedures recorded at Rochester medical facilities are continuously indexed for automated retrieval [21, 22]. The medical record includes the detailed history of all medical encounters in the community including ambulatory medical and social services, hospitals, emergency departments, and home visits, as well as laboratory, psychiatry, and psychology reports and test results. The study was approved by the Institutional Review Boards of Mayo Clinic and Olmsted Medical Center.

### Birth cohort

Our birth cohort consisted of all children born between January 1, 1976 and December 31, 1982 to mothers residing in the townships comprising Minnesota Independent School District No. 535 (n = 8548). The target population consisted of 5718 children (2956 boys and 2762 girls) who still lived in Rochester at or after the age of five years [23, 24], who were followed retrospectively (i.e., whose medical records were abstracted) from birth until the occurrence of either death or last clinical follow-up until 19 years of age). The steps and resources used for identification and follow-up of this birth cohort, and analysis of potential influence of migration bias, have been reported [25].

### ADHD incidence cases-identification and case definition

Our strategy in identifying ADHD incidence cases consisted of several steps, used multiple sources of information and relied on recorded history of symptoms, individual test results, and treatment. The details of this process were previously described [24]. In short, several steps were used to identify potential ADHD incidence cases, starting with cumulative school records of each child in the birth cohort (n = 5718). School records were searched for any indication of concerns about learning and behavior, and 1961 children had those concerns observed by teachers, parents, school psychologists, physicians, social workers, and school nurses. Further work on these 1961 children consisted of abstracting data from the school and medical records and the records from the only private psychiatric practice in the community.

Identification of ADHD incidence cases consisted of applying research criteria to the 1961 children from our birth cohort who had any recorded behavioral or learning concerns. Subjects were defined as research-identified ADHD incidence cases if their school and/or medical records included various combinations of the following three different categories of information: 1) met DSM-IV criteria for ADHD, 2) positive ADHD questionnaire results, 3) clinical diagnosis of ADHD documented. Details of information regarding those criteria as well as identification process of ADHD cases were described elsewhere [24]. Through this research protocol, a total of 379 ADHD cases were identified.

### Non-ADHD controls

For each of the 379 ADHD incident cases, two control subjects were selected from the birth cohort. The controls were matched individually on gender and date of birth (±6 months) using an optimal matching algorithm applied to the values of the two matching factors [24]. None of the controls had a diagnosis of pervasive developmental disorder, severe intellectual disability, schizophrenia, or any psychotic disorder.

### Psychiatric disorders-cases identification

The procedure employed to identify all comorbid psychiatric disorders among ADHD cases and controls prior to 19 years of age consisted of a systematic, multi-staged process, utilizing detailed, routinely collected data as part of the Rochester Epidemiology Project resources [21]. First, a list of all psychiatric diagnoses among all ADHD cases and controls was systematically produced using a computerized medical index retrieval system that is an integral component of the Rochester Epidemiology Project [21]. It should be noted that, as the study extended up to age 19 years, many different terms were used over time to describe similar groupings of psychiatric conditions (e.g., depression, neurotic depression, etc.). Second, members of the research team (two psychologists, two developmental-behavioral pediatricians and a physician epidemiologist) individually reviewed this list, and it was determined by consensus that among possible psychiatric diagnoses, 299 diagnosis codes could be classified as psychiatric disorders in one or more versions of the Diagnostic and Statistical Manual of Mental Disorders. Third, we abstracted the medical records for all ADHD cases and controls who had at least one of these 299 diagnoses before age 19 years. To be classified with a psychiatric disorder, we required documentation of (a) the initial diagnosis and (b) separate confirmation of the initial diagnosis. Documented diagnoses were considered to have been confirmed when the confirmatory diagnosis was made by a qualified professional with appropriate credentials and expertise. Trained abstractors, blinded for ADHD case/control status, collected the following information: psychiatric diagnosis, earliest date of diagnosis, professional making the initial psychiatric diagnosis, date of confirmation and by whom (medical professional).

These 299 psychiatric diagnoses were collapsed into 10 categories based on their classification in the DSM-IV (comparable terms remain in DSM-5) [26]. Thus, we combined the diagnoses of ‘depressive disorder’ and ‘major depressive disorder,’ since the number with major depressive disorder was extremely small. Terms such as ‘depressed mood’, ‘depression’, ‘dysthymia’, and ‘mood disorder’ were all classified as “depressive disorder”. Bipolar disorder was excluded from this category. Diagnosis of anxiety disorders included disorders listed in DSM-IV such as panic attack, agoraphobia, specific or social phobia, obsessive-compulsive disorders, post-traumatic stress disorder, and generalized anxiety disorder. Diagnosis of SUD included the diagnoses related to abuse or dependence of any kind of substance listed in DSM-IV, such as alcohol, amphetamine, cannabis, marijuana, cocaine, LSD, and so on. More than 80% of children with these psychiatric disorders (i.e., depressive disorder, anxiety disorder, CD/ODD and SUD) were diagnosed by psychologists or psychiatrists. Other clinicians such as internists, pediatricians, family doctors, and masters of psychology who could make psychiatric diagnoses also made such diagnoses.

### Statistical analysis

Analyses were performed using the SAS version 9.3 software (SAS Institute, Inc.; Cary, NC). For each subject the duration of follow-up was calculated from birth until SUD diagnosis prior to 19 years of age; otherwise, for subjects without an SUD diagnosis prior to 19 years of age, their follow-up duration was censored at the date of last clinical follow-up if the subject was last seen prior to 19 years of age, or at age 19 if last seen after age 19. The association between ADHD case status and SUD was evaluated using Cox proportional hazards models; the association was summarized using the hazard ratio (HR) and corresponding 95% confidence interval (CI) derived from the parameters estimates. Separate analyses were performed to evaluate the moderating and mediating effect of each of three psychiatric disorders (depression, CD/ODD, anxiety) on the association between ADHD and SUD. The presence or absence of each psychiatric disorder was handled using a time-dependent covariate and the start/stop counting process style of the Cox model [27]; therefore, only psychiatric disorders (depression, CD/ODD, anxiety) diagnosed before the SUD diagnosis date were considered in the analysis. ADHD status was handled as a standard covariate and thereby assumed to predate the other psychiatric disorders and SUD. Each analysis consisted of three steps, patterned after the analysis by Tuithof et al. [28] and following the recommendations for presenting analyses of effect modification and interaction by Knol and VanderWeele [29].

First, an assessment of a moderating or interaction effect of the psychiatric disorder on an additive scale was performed by evaluating the joint effects of ADHD and the psychiatric disorder in a Cox model using the methods outlined by Li and Chambless [30]. The relative excess risk due to interaction (RERI) was calculated as RERI = HR_AB_−HR_A_−HR_B_ +1, where HR_AB_, HR_A_, and HR_B_ are the corresponding HRs for those with both ADHD and the psychiatric disorder, ADHD only, and the psychiatric disorder only, respectively [31]. In the absence of an additive interaction effect, the RERI would be 0. Second, a test for the moderating or interaction effect on a multiplicative scale was conducted by adding a product term to the Cox model for the product between ADHD case status and the time-dependent covariate for the presence/ absence of the psychiatric disorder. Contrast statements in SAS Proc PHREG were utilized to obtain stratum-specific HRs for subjects with and without the psychiatric disorder. In the absence of a multiplicative interaction effect, the ratio of the two stratum-specific HRs would be 1. Finally, the mediating effect of the psychiatric disorder on the association between ADHD and SUD was evaluated by adding a time-dependent covariate for the presence/ absence of the psychiatric disorder to a Cox model that included a covariate for ADHD case status. The Cox models were fit with and without adjusting for subject gender, maternal age, maternal education (less than high school education, high school graduate, some college, college graduate, unknown), and marital status at birth.

## Results

Among the 5718 subjects in the birth cohort, 19 children with severe intellectual disability were excluded, leaving 5699 subjects. A total of 379 children fulfilled the research criteria for ADHD at a mean (SD) age of 10.4 (3.6) years (median 9.8 years). The study initially included 1137 subjects: 379 ADHD cases each with two gender and age matched controls (n = 758). However, 36 cases and 46 controls were excluded from the analysis because they denied access to their medical records for research purposes. The analyses herein are based on the remaining 343 ADHD cases and 712 non-ADHD controls. A comparison of demographic and perinatal factors between cases and controls has been previously described [5], indicating no significant differences regarding these factors between these groups. Furthermore, as previously reported [5], a total of 68 ADHD cases and 41 non-ADHD controls had a SUD by 19 years of age, and ADHD was significantly associated with an increased risk of SUD by age 19 years (HR = 3.70, 95% CI = 2.51 to 5.46). The strength of the association was not altered after adjusting for gender, maternal age, maternal education and maternal marital status (adjusted HR = 3.64, 95% CI = 2.46 to 5.38).

Table 1 summarizes the effect of depression on the association between ADHD and SUD. Compared to subjects without depression and without ADHD, subjects who only had ADHD were at a higher risk to develop SUD (adjusted HR = 3.85). Those who had depression only were also at a higher risk (adjusted HR = 9.07) while those who had both depression and ADHD had a slightly higher risk (adjusted HR = 9.94) to develop SUD. Therefore, based on the analysis of the joint effects, the data did not support an additive interaction effect, as the combined effect of ADHD and depression (adjusted HR = 9.94) was not stronger than the sum of the separate effects of ADHD only or depression only, and the relative excess risk due to interaction was not significantly different from 0 (RERI = -1.98, 95% CI = -9.51 to 5.54, p = 0.61). Furthermore, on a multiplicative scale, there was a negative or antagonistic interaction between ADHD and depression (ratio of stratum-specific adjusted HRs = 0.28, 95% CI = 0.12 to 0.70, p = 0.007). In particular, among those with depression, there was no statistically significant difference in the risk for SUD between those with and without ADHD (adjusted HR = 1.10, p = 0.82), while among those without depression, subjects with ADHD were more likely to develop SUD (adjusted HR = 3.85, p<0.001). Lastly, the association between ADHD and SUD was slightly attenuated after adjusting for depression (adjusted HR = 3.64 decreased to HR = 2.95, 95% CI = 1.97 to 4.41), indicating that the association between ADHD and SUD was partially, but not fully, mediated by depression.

**Table 1:** Evaluation of the role of depression on the association between ADHD and SUD.

	Unadjusted		Adjusted ^a^	
	HR (95% CI)	P	HR (95% CI)	P
**Joint effects**				
No Depression, No ADHD	1.00		1.00	
No Depression, ADHD	4.03 (2.58, 6.31)	<0.001	3.85 (2.45, 6.04)	<0.001
Depression, No ADHD	8.59 (4.19, 17.61)	<0.001	9.07 (4.40, 18.69)	<0.001
Depression, ADHD	9.44 (5.21, 17.11) ^b^	<0.001	9.94 (5.45, 18.12) ^c^	<0.001
**Stratum-specific effects** ^d^				
Depression: ADHD vs. Non-ADHD	1.10 (0.50, 2.40)	0.81	1.10 (0.50, 2.40)	0.82
No Depression: ADHD vs. Non-ADHD	4.03 (2.58, 6.31)	<0.001	3.85 (2.45, 6.04)	<0.001

^a^ Multivariable model adjusted for gender, maternal age, maternal education, and maternal marital status at the subject’s birth.

^b^ The expected HR under the assumption of additivity is 11.62. This is contained within the 95% CI for the observed combined effect (95% CI, 5.21, 17.11), so therefore there is not sufficient evidence to suggest a moderating effect on an additive scale. Likewise the combined effect of ADHD and depression is not significantly higher than the sum of the separate effects of ADHD only and depression only.

^c^ The expected HR under the assumption of additivity is 11.92. This is contained within the 95% CI for the observed combined effect (95% CI, 5.45, 18.12), so therefore there is not sufficient evidence to suggest a moderating effect on an additive scale. Likewise the combined effect of ADHD and depression is not significantly higher than sum of the separate effects of ADHD only and depression only.

^d^ On a multiplicative scale, there is a negative or antagonistic interaction between ADHD and depression (ratio of stratum-specific adjusted HRs = 0.28, 95% CI = 0.12 to 0.70, p = 0.007). In particular, among those with depression, there is not a statistically significant difference in the risk for SUD between those with and without ADHD (adjusted HR = 1.10), while among those without depression, subjects with ADHD are more likely to develop SUD (adjusted HR = 3.85).

The same statistical analyses were conducted for CD/ODD and results are shown in Table 2. Compared to subjects without CD/ODD and without ADHD, those who only had ADHD were at higher risk to develop SUD (adjusted HR = 3.55). Those who had only CD/ODD were also at higher risk (adjusted HR = 5.86) to develop SUD. In addition, those who had both CD/ODD and ADHD also had high risk (adjusted HR = 5.76) to develop SUD. Based on the above analysis of the joint effects, the data did not support an additive interaction effect (RERI = -2.65, 95% CI = -9.16 to 3.87, p = 0.43). However, on a multiplicative scale, there was a negative or antagonistic interaction between ADHD and CD/ODD (ratio of HRs = 0.28, 95% CI = 0.09 to 0.89, p = 0.031); among those with CD/ODD, there was no statistically significant difference in the risk for SUD between those with and without ADHD (adjusted HR = 0.98, p = 0.98), yet among those without CD/ODD, subjects with ADHD were more likely to develop SUD (adjusted HR = 3.55, p<0.001). Just like depression, the association between ADHD and SUD was partially mediated by CD/ODD (adjusted HR = 3.64 decreased to 3.14, 95% CI = 2.08 to 4.76).

**Table 2:** Evaluation of the role of CD/ODD on the association between ADHD and SUD.

	Unadjusted		Adjusted ^a^	
	HR (95% CI)	P	HR (95% CI)	P
**Joint effects**				
No CD/ODD, No ADHD	1.00		1.00	
No CD/ODD, ADHD	3.54 (2.31, 5.43)	<0.001	3.55 (2.31, 5.46)	<0.001
CD/ODD, No ADHD	6.46 (2.30, 18.13)	<0.001	5.86 (2.07, 16.57)	<0.001
CD/ODD, ADHD	6.34 (3.65, 11.03) ^b^	<0.001	5.76 (3.26, 10.19) ^c^	<0.001
**Stratum-specific effects** ^d^				
CD/ODD: ADHD vs. non-ADHD	0.98 (0.33, 2.89)	0.97	0.98 (0.33, 2.91)	0.98
No CD/ODD: ADHD vs. non-ADHD	3.54 (2.31, 5.43)	<0.001	3.55 (2.31, 5.46)	<0.001

^a^ Multivariable model adjusted for gender, maternal age, maternal education, and maternal marital status at the subject’s birth.

^b^ The expected HR under the assumption of additivity is 9.00. This is contained within the 95% CI for the observed combined effect (95% CI, 3.65, 11.03), so therefore there is not sufficient evidence to suggest a moderating effect on an additive scale. Likewise the combined effect of ADHD and CD/ODD is not significantly higher than the sum of the separate effects of ADHD only and CD/ODD only.

^c^ The expected HR under the assumption of additivity is 8.41. This is contained within the 95% CI for the observed combined effect (95% CI, 3.26, 10.19), so therefore there is not sufficient evidence to suggest a moderating effect on an additive scale. Likewise the combined effect of ADHD and CD/ODD is not significantly higher than sum of the separate effects of ADHD only and CD/ODD only.

^d^ On a multiplicative scale, there is a negative or antagonistic interaction between ADHD and CD/ODD (ratio of stratum-specific adjusted HRs = 0.28, 95% CI = 0.09 to 0.89, p = 0.031). In particular, among those with CD/ODD, there is not a statistically significant difference in the risk for SUD between those with and without ADHD (adjusted HR = 0.98), while among those without CD/ODD, subjects with ADHD are more likely to develop SUD (adjusted HR = 3.55).

In the same manner, Table 3 summarizes the role of anxiety on the association between ADHD and SUD. The moderating effect of anxiety was not statistically significant on either the additive scale (RERI = 2.20, 95% CI = -3.23 to 7.62, p = 0.43) or the multiplicative scale (ratio of HRs = 1.38, 95% CI = 0.16 to 11.96, p = 0.77). As with depression and CD/ODD, the association between ADHD and SUD was slightly attenuated after adjusting for anxiety (adjusted HR = 3.64 decreased to 3.55, 95% CI 2.40 to 5.26).

**Table 3:** Evaluation of the role of anxiety on the association between ADHD and SUD.

	Unadjusted		Adjusted ^a^	
	HR (95% CI)	P	HR (95% CI)	P
**Joint effects**				
No Anxiety, No ADHD	1.00		1.00	
No Anxiety, ADHD	3.61 (2.42, 5.37)	<0.001	3.51 (2.35, 5.24)	<0.001
Anxiety, No ADHD	1.26 (0.17, 9.20)	0.82	1.22 (0.17, 8.95)	0.84
Anxiety, ADHD	5.59 (2.37, 13.20) ^b^	<0.001	5.93 (2.50, 14.04) ^c^	<0.001
**Stratum-specific effects** ^d^				
Anxiety: ADHD vs. non-ADHD	4.42 (0.53, 36.73)	0.17	4.84 (0.58, 40.38)	0.14
No Anxiety: ADHD vs. non-ADHD	3.61 (2.42, 5.37)	<0.001	3.51 (2.35, 5.24)	<0.001

^a^ Multivariable model adjusted for gender, maternal age, maternal education, and maternal marital status at the subject’s birth.

^b^ The expected HR under the assumption of additivity is 3.87. This is contained within the 95% CI for the observed combined effect (95% CI, 2.37, 13.20), so therefore there is not sufficient evidence to suggest a moderating effect on an additive scale. Likewise the combined effect of ADHD and anxiety is not significantly higher than the sum of the separate effects of ADHD only and anxiety only.

^c^ The expected HR under the assumption of additivity is 3.73. This is contained within the 95% CI for the observed combined effect (95% CI, 2.50, 14.04), so therefore there is not sufficient evidence to suggest a moderating effect on an additive scale. Likewise the combined effect of ADHD and anxiety is not significantly higher than the sum of the separate effects of ADHD only and anxiety only.

^d^ On a multiplicative scale, there is a not a significant interaction between ADHD and anxiety (ratio of stratum-specific adjusted HRs = 1.38, 95% CI = 0.16 to 11.96, p = 0.77); the magnitude of the association between ADHD and SUD is similar among those with and without anxiety.

## Discussion

Population-based, non-referred samples of adolescents with ADHD and SUD are of critical importance in order to increase our understanding of the natural history of the comorbidity between ADHD and SUD. The core of this epidemiologic study is the population-based sample of carefully defined, research-identified ADHD cases and non-ADHD controls, as well as the standardized procedures for ascertaining SUD and the three common internalizing and externalizing psychiatric disorders. Our previous report, using multivariate analyses, from this population-based birth cohort clearly indicates a strong association between ADHD and SUD [5]. However, to understand the underlying mechanism of the significant association between ADHD and SUD requires further investigation. Existing evidence suggests that combined environmental and genetic factors are involved in the pathogenesis of the comorbidity between these disorders [32]. Considering shared genetic factors between ADHD and SUD [33, 34], the association between these two disorders might be explained by the correlated liability hypothesis, which states that an increase in liability for one disorder is correlated with an increase in liability for the second disorder [35]. Our study, however, suggests that perhaps comorbid depression or CD/ODD, rather than ADHD itself, links ADHD with SUD. In addition, dual diagnoses of typical externalizing (e.g., CD/ODD) and internalizing disorders (depression, anxiety) among ADHD patients with SUD are common [36, 37]. This might suggest that the adverse effect of ADHD on SUD could be magnified by such comorbidities.

To better understand the clinical implications of the three common psychiatric comorbid conditions, we evaluated the effect of depression, CD/ODD and anxiety on the association between ADHD and SUD. Molina and Pelham [15] showed that persistence of ADHD and adolescent CD was associated with an elevated risk of SUD. Fergusson et al. [16] also showed that conduct problems in childhood and adolescence were related to later substance use after controlling for attention problems. Conversely, attention problems were unrelated to later substance use after controlling for conduct problems. These findings suggest that CD is more strongly associated with SUD than ADHD alone [16], consistent with our findings.

Analyses based on moderating and mediating models have been shown to be important tools in developmental and behavioral pediatric research [19]. Compared to subjects with neither CD/ODD nor ADHD, Tuithof et al. [30], using moderating and mediating models, showed that CD alone is more strongly associated with an increased risk of SUD than ADHD alone, results that are consistent with our findings.

We found that depression was also more strongly associated with an increased risk of SUD than ADHD alone. Major depression or dysthymia was reported by Williams et al. to be more strongly associated with substance use than ADHD (odds ratio = 4.0 vs. 2.7) among adolescents of whom 66% were HIV positive, consistent with our findings [38].

Unexpectedly, we found that adolescents with both depression (or CD/ODD) and ADHD were not at much greater risk to develop SUD than those with depression only (or CD/ODD only). Depression may motivate affected individuals to use substances, and CD/ODD is linked with delinquency, which in turn is strongly associated with the substance use that our results demonstrated. These disorders themselves might be strong risk factors leading to SUD compared to ADHD [39]. Consequently, adverse effects of ADHD on SUD might be somewhat obscured in individuals with ADHD and co-morbid depression or CD/ODD. This is supported by the negative or antagonistic interaction between ADHD and depression or CD/ODD on SUD observed in our study. More specifically, the combined effect of ADHD and either of these psychiatric disorders did not exceed the sum of the effects taken independently of each other.

Our further analyses showed that the association between ADHD and SUD is only slightly mediated by such comorbid disorders. Other studies have reported the mediating effects of CD/ODD on the association between ADHD and SUD. For example, in the meta-analysis by Serra-Pinheiro et al. [40], 15 studies presented odds ratios (OR) for the development of illicit substance use in patients with ADHD controlling for CD/ODD. Their results suggested that ADHD was not significantly associated with illicit substance use after adjusting for CD/ODD (summary OR = 1.35, 95% CI 0.9–2.0) [40]. Among these 15 studies, 11 included only adolescents, while the other four included adults. Nine studies utilized a longitudinal study design, while the others utilized a cross-sectional design (one study used both prospective and cross-sectional design) [40]. Though the results of this meta-analysis support the mediating role of CD on the association between ADHD and SUD, due to such large heterogeneity among the studies (I^2^ = 55%), it might be difficult to compare these results directly with our findings. The authors of this meta-analysis also reported that the failure to detect a significant association might be due to statistical power issues stemming from small numbers of ADHD cases in each study [40]. In addition, two other studies [30, 41] also reported that ADHD was not significantly associated with an increased risk of SUD after controlling for CD, supporting our findings.

Limitations should be considered when interpreting the present results. First, this is a retrospective cohort study dependent on retrospective review of medical and school records; therefore, the precise age of onset of ADHD could not be determined. Since the etiology of ADHD is determined by complex effects of prenatal, perinatal, and genetic factors [42], ADHD status was assumed to predate other psychiatric disorders. Second, even though we statistically demonstrated a significant mediator and moderator effect (on a multiplicative scale) of depression and CD/ODD on the association between ADHD and SUD this does not establish etiology (causality) between these disorders. Third, it is possible that some comorbid psychiatric disorders were not identified. However, multiple independent complementary sources of data were used for the identification of these disorders. Further, diagnostic category of anxiety disorders in DSM-IV is too heterogeneous and significant effects of one type of anxiety disorder might have been obscured by null or opposite effects of others. Finally, Rochester is primarily a white, middle class community, which may limit the generalizability of these results. Nevertheless, these data provide much needed information for comparison with similar studies [43, 44].

In conclusion, while our current findings confirmed our previous work and work of others showing a significant association between ADHD and substance-related disorders in adolescents [5, 45], our current study suggests that depression and CDD/OD may be just as, or even more, important precursors of substance dependence or abuse compared to ADHD. While these findings do not diminish the importance of careful monitoring for SUD among children and adolescents with ADHD, our findings highlight the need to also carefully monitor adolescents with depression or CD/ODD for SUD prevention and treatment, regardless of whether ADHD is present. Furthermore, basic research is needed to elucidate this relationship.
